# A Novel Triglyceride- and Cholesterol-Enriched Lipoprotein(a) Phenotype in Moderate Hypertriglyceridemia: Reversal by ApoC-III Inhibition

**DOI:** 10.1016/j.jacbts.2026.101699

**Published:** 2026-09-17

**Authors:** Lizhu Lin, Maiah N. Brush, C. Bradley Nelson, Veronica J. Alexander, Shuting Xia, Philip L.S.M. Gordts, Sotirios Tsimikas

**Affiliations:** a Division of Cardiovascular Medicine, Department of Medicine, University of California, San Diego, La Jolla, California, USA; b University of Utah Molecular Medicine Program, Salt Lake City, Utah, USA; c Division of Microbiology and Immunology, Department of Pathology, University of Utah School of Medicine, Salt Lake City, Utah, USA; d Ionis Pharmaceuticals, Carlsbad, California, USA; e Division of Endocrinology and Metabolism, Department of Medicine, University of California, San Diego, La Jolla, California, USA

**Keywords:** ApoC-III, cholesterol, lipoprotein(a), triglycerides

## Abstract

In moderate hypertriglyceridemia, lipoprotein(a) (Lp[a]) particles were enriched in triglycerides and cholesterol, accounting for about 16% to 21% of plasma triglycerides and about 9% to 11% of plasma cholesterol. In a phase 2 trial, apolipoprotein C-III inhibition with olezarsen reduced Lp(a)-associated triglycerides and cholesterol by about 56% to 70%, with no consistent reduction in plasma Lp(a). Oxidized phospholipid measures tracked more closely with Lp(a) particle concentration than with Lp(a)-associated lipid content, supporting partially distinct dimensions of Lp(a) biology. These findings identify Lp(a) lipid composition as a dynamic, therapeutically modifiable feature in hypertriglyceridemia. (Study of ISIS 678354 [AKCEA-APOCIII-LRx] in Participants With Hypertriglyceridemia and Established Cardiovascular Disease [CVD]; NCT03385239)

## Highlights

• Moderate hypertriglyceridemia was associated with TG- and cholesterol-enriched Lp(a).
• Olezarsen reduced Lp(a)-TG and Lp(a)-C by 60–70% without changing particle number.
• OxPL correlated with Lp(a), but not Lp(a)-TG, supporting distinct Lp(a) traits.
• FPLC selectively depleted TG-rich, apoC-III–containing Lp(a) particles.
• These findings identify a modifiable Lp(a) subspecies for cardiovascular risk.

## Visual Abstract

**Figure:**
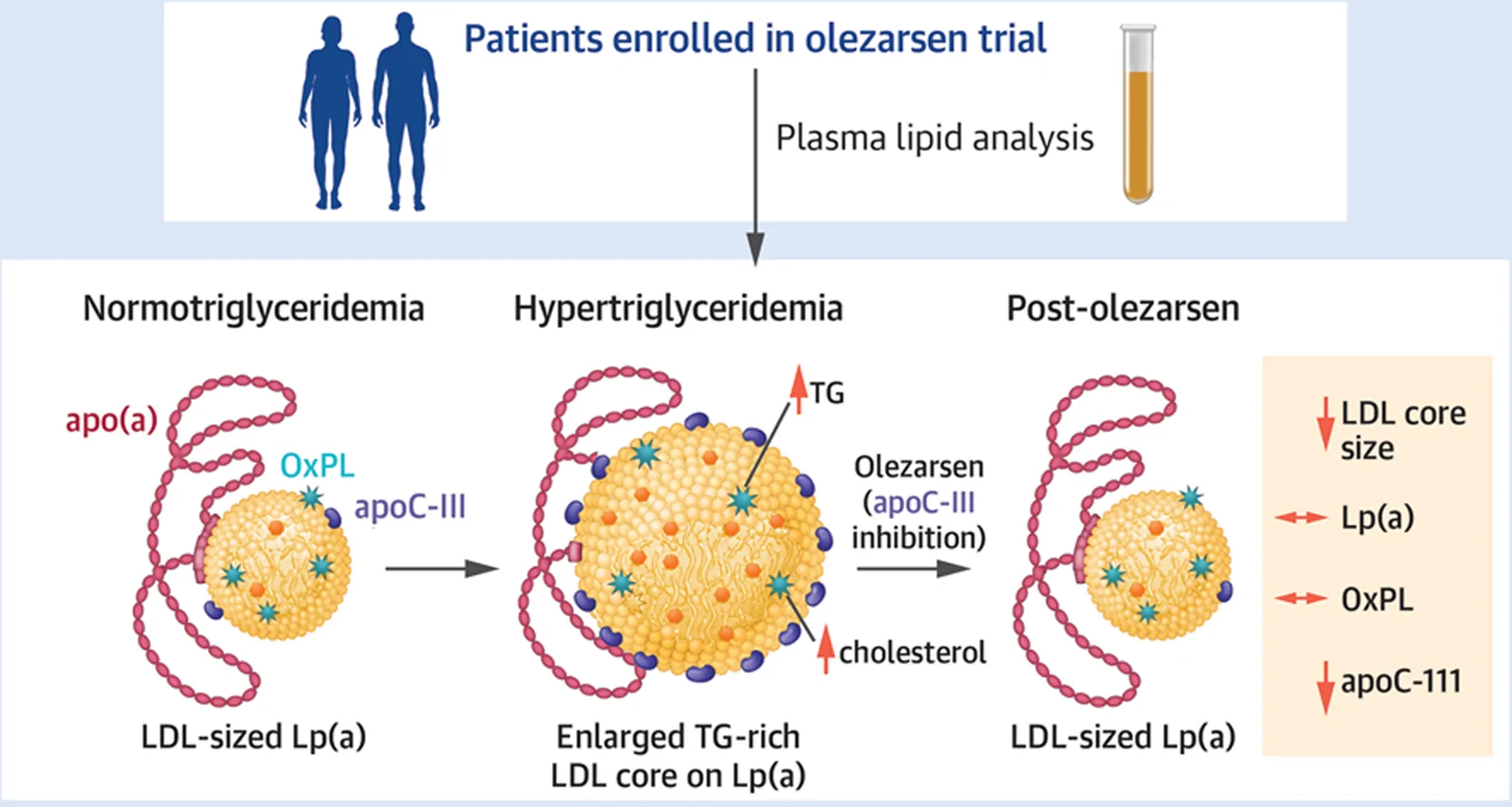

Lipoprotein(a) (Lp[a]) is a genetically determined cardiovascular risk factor typically characterized by particle concentration rather than particle composition.^1^^,^^2^ Whether Lp(a) lipid composition is altered in dyslipidemic states, and whether such alterations are reversible, remains poorly defined in humans. Lp(a) consists of the hydrophilic, non-lipid-containing apolipoprotein(a) (apo[a]) covalently bound to the hydrophobic, lipid-containing apolipoprotein B-100 (apoB), which surrounds a lipid core rich in cholesteryl esters and triglycerides, with free cholesterol and phospholipids on the particle surface.^3^

Lp(a) is typically referred to as “low-density lipoprotein (LDL)–like,” as it generally has the density and size most typical of LDL particles in normolipidemic individuals.^4^^,^^5^ However, in epidemiologic studies, a modest inverse (*r* = −0.10 to 0.20) correlation exists between plasma levels of Lp(a) and triglycerides,^6^, ^7^, ^8^, ^9^ suggesting a pathophysiological relationship between Lp(a) and triglyceride metabolism. These observations, however, are limited to particle concentration and do not address whether the lipid composition of Lp(a) particles is altered in hypertriglyceridemic states. In normolipidemic individuals, Lp(a) contains relatively little triglyceride, typically <5% of total lipid mass. In contrast, studies in moderate hypertriglyceridemia suggest that 15% to 20% of apo(a) immunoreactivity is present in the very low density lipoprotein (VLDL) density range.^10^, ^11^, ^12^ Whether such changes are dynamic and reversible with triglyceride-lowering therapy remains uncertain.^13^

Apolipoprotein C-III (apoC-III) is a key determinant of triglyceride-rich lipoprotein (TRL) metabolism, as it inhibits lipoprotein lipase–mediated size remodeling of TRLs and the hepatic uptake of intact remnant TRL particles via the canonical TRL clearance receptors LDLR and LRP1.^14^, ^15^, ^16^ The presence of apo(a) on LDL particles impedes effective hepatic particle clearance via the same receptors.^17^ Olezarsen, an antisense oligonucleotide targeting apoC-III, reduces plasma triglycerides and remnant lipoproteins and is approved for treatment of familial chylomicronemia syndrome.^18^, ^19^, ^20^, ^21^, ^22^, ^23^

We therefore sought to determine whether Lp(a) is enriched in triglycerides and cholesterol in moderate hypertriglyceridemia and whether apoC-III inhibition reverses this phenotype. Using validated immune isolation assays and lipoprotein fractionation, we examined Lp(a) compositional changes in participants treated with olezarsen.

## Methods

### Study population and study design

This was a substudy of a prior phase 2, randomized, double-blind, placebo-controlled, dose-ranging study evaluating olezarsen, an N-acetyl-galactosamine-conjugated antisense oligonucleotide targeted to hepatic *APOC3* messenger RNA to inhibit apoC-III production in patients at high risk for or with established cardiovascular disease.^19^ Briefly, this study was conducted in 114 patients with fasting serum triglycerides 200 to 500 mg/dL (2.26-5.65 mmol/L). Patients received olezarsen (10 or 50 mg every 4 weeks, 15 mg every 2 weeks, or 10 mg every week) or saline placebo subcutaneously for 6 to 12 months. These regimens correspond to cumulative monthly doses of 10, 30, 40, and 50 mg, respectively. The primary endpoint was the percentage change in fasting triglyceride levels from baseline to month 6 (mean data of weeks 25-27) of exposure.

For the present analysis, blood samples were evaluated at baseline, week 17, and week 25 for patients treated every 4 weeks and week 27 for patients every week or every 2 weeks. The demographic, clinical, and laboratory characteristics and main findings of this study were previously described.^19^

The protocol was approved by the Institutional Review Board at each participating center and by an ethics committee (Quorum Review Institutional Review Board), and the trial was performed in accordance with the principles of the Declaration of Helsinki and current Good Clinical Practice guidelines. All patients provided written informed consent before enrollment.

### Quantification of Lp(a)-associated triglycerides and Lp(a)-associated cholesterol and Lp(a) particle concentration

Detailed analytical validation of the immune isolation assays used to quantify Lp(a)-associated triglycerides (Lp[a]-TG) is reported separately.^13^ In brief, Lp(a) is selectively isolated from plasma using magnetic beads conjugated with monoclonal antibody LPA4 targeting apo(a),^24^ followed by enzymatic quantification of triglycerides. Assay specificity was ensured using disruption buffers to prevent nonspecific lipoprotein interactions. Spike-in experiments with purified VLDL or intermediate-density lipoprotein lacking Lp(a) demonstrated no measurable interference. In 36 normotriglyceridemic individuals, mean plasma triglycerides were 98.4 ± 31.9 mg/dL, with mean Lp(a)-TG of 1.42 ± 2.83 mg/dL, mean Lp(a)-associated cholesterol (Lp[a]-C) of 4.03 ± 4.01 mg/dL, and a low Lp(a)-TG/Lp(a)-C ratio of 0.59 ± 1.27, consistent with a predominantly LDL-like Lp(a) phenotype.^13^

Lp(a)-C^25^^,^^26^ was quantified using a validated immune isolation assays. Lp(a) molar concentration was measured with the isoform insensitive Roche TinaQuant assay calibrated to nanomoles per liter (Medpace Research Laboratories).

### Fast protein liquid chromatography

Lipoprotein fractionation of human plasma samples (0.1 mL) was performed using fast protein liquid chromatography (FPLC). Plasma samples were obtained for 5 patients treated with placebo and 5 patients treated with olezarsen 50 mg subcutaneously monthly at baseline prior to treatment and at 6 months of treatment. For FPLC, samples were obtained from participants receiving the highest olezarsen exposure, corresponding to a 50-mg monthly dose. The baseline and 6-month lipid variables of these study subjects are shown in Supplemental Table 1. The plasma samples were loaded on a GE Superose 6 10/30 GL column, and fractions corresponding to VLDL, intermediate-density lipoprotein, LDL, and Lp(a)-containing lipoproteins were identified on the basis of elution profiles and confirmed using lipid and apolipoprotein measurements. The late-eluting, nonlipoprotein fraction (approximately fractions 52-60) contains free glycerol and other small, water-soluble metabolites generated during intravascular triglyceride hydrolysis. Triglycerides and cholesterol were quantified in each fraction using enzymatic assays (Pointe Scientific).

### Immunoassays to measure intact Lp(a), total apo(a), apoC-III-apo(a) and apolipoprotein E–apo(a) complexes

The presence of intact Lp(a) (defined as apo[a] covalently bound to apoB), total apo(a) (free apo[a] plus apo[a] covalently bound to apoB-100), and apo(a) complexes with apoC-III and apolipoprotein E (apoE) were quantified in FPLC fractions using antibody-based immunoassays. Intact Lp(a) was measured using a sandwich immunoassay with capture by the apoB-100-specific monoclonal antibody MB47 (5 μg/mL)^27^^,^^28^ and detection with biotinylated LPA4 (1 μg/mL), as previously described.^24^ Total apo(a) was measured using LPA4 (5 μg/mL) as both the capture antibody and biotinylated detection antibody (1 μg/mL). ApoC-III-apo(a) complexes were detected using LPA4 (5 μg/mL) as the capture antibody and a rabbit monoclonal antibody against human apoC-III (ab76305, Abcam; 1 μg/mL) for detection. ApoE-apo(a) complexes were detected using LPA4 (5 μg/mL) as the capture antibody and a rabbit polyclonal antibody recognizing all human apoE isoforms (ab308649, Abcam; 1 μg/mL) as the detection antibody. Changes in lipoprotein distribution before and after 6 months of olezarsen treatment (50 mg monthly) were assessed by comparing fractional lipid and apolipoprotein profiles across FPLC fractions. To evaluate TRL- versus non-TRL-associated variables, the relative light units (RLU) measured across FPLC fractions 1 to 15 were summed and designated as the TRL-associated fraction, whereas the RLU measured across fractions 16 to 70 were summed and designated as the non-TRL-associated fraction. The relative distribution of each variable between the TRL and non-TRL fractions was subsequently calculated as the percentage of the total RLU detected across fractions 1 to 70.

### Derived ratios and compositional indexes

To assess compositional remodeling of Lp(a), the ratio of Lp(a)-TG to Lp(a)-C was calculated using mass concentrations. Additional lipid-to-particle ratios were derived by normalizing for Lp(a) molar concentration: Lp(a)-TG/Lp(a) molar ratio and Lp(a)-C/Lp(a) molar ratio, enabling the assessment of lipid content per Lp(a) particle.

### Measurement of oxidized phospholipids on lipoproteins (apoB-100, apo[a], apoC-III, and apolipoprotein AI)

**Figure 1:**
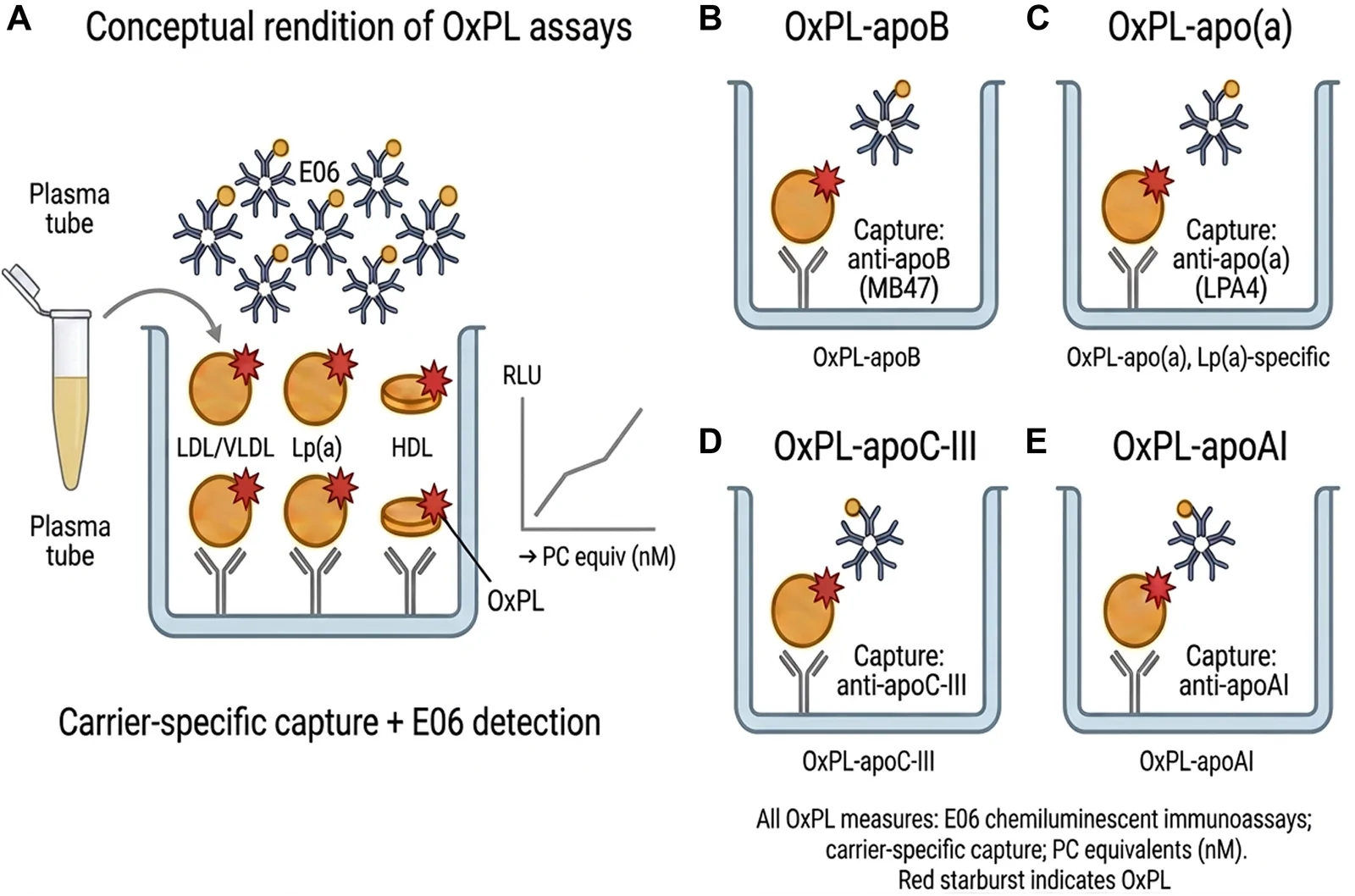
Methodology of OxPL Immunoassays on ApoB, Apo(a), ApoC-III, and ApoAI Lipoprotein Carriers (A) Overview of chemiluminescent assays in which plasma is added to wells coated with carrier-specific capture antibodies, allowing immobilization of low-density lipoprotein (LDL) or very low density lipoprotein (VLDL), lipoprotein(a) (Lp[a]), or high-density lipoprotein (HDL) particles; oxidized phospholipids (OxPL; red starburst) on captured particles are detected by biotinylated E06 immunoglobulin M, and signal is reported as relative light units (RLU) converted to phosphocholine (PC) equivalents (equiv). (B-E) Individual assays for OxPL–apolipoprotein B (apoB), OxPL–apolipoprotein(a) (apo[a]), OxPL–apolipoprotein C-III (apoC-III), and OxPL–apolipoprotein AI (apoAI), respectively, using capture antibodies to apoB (MB47), apo(a) (LPA4), apoC-III, and apoAI to isolate the corresponding lipoprotein carrier before detection with E06.

Oxidized phospholipids (OxPL) associated with apoB (OxPL-apoB), apo(a) (OxPL-apo[a]),^29^ and apolipoprotein AI (OxPL-apoAI)^30^ were quantified using established chemiluminescent immunoassays, as previously described. To assess the role of OxPL in triglyceride metabolism, a novel assay was developed to measure OxPL on apoC-III-containing particles (OxPL-apoC-III) (Figure 1).

In all assays, apolipoprotein-specific capture antibodies were immobilized on microtiter plates to isolate the corresponding lipoprotein fraction. Bound OxPL were detected using biotinylated immunoglobulin M monoclonal antibody E06, which recognizes the phosphocholine headgroup of oxidized, but not native, phospholipids. Signals were quantified by chemiluminescence and expressed as RLU.

For the OxPL-apoC-III assay, a rabbit monoclonal anti-apoC-III antibody (2 μg/mL; Abgent) was used for capture with documentation of a saturating amount of antibody, as previously described for measuring total apoC-III levels.^31^ Calibration was performed using phosphocholine–bovine serum albumin, and results were expressed as nanomolar phosphocholine equivalents. Intra- and interassay coefficients of variation were <10%. Baseline and longitudinal samples from each participant were analyzed within the same assay run to minimize variability.

### Statistical analyses

Laboratory data are presented as mean ± SD or median (Q1-Q3), unless otherwise specified. The analysis of percentage changes was conducted using a mixed-effects model for repeated measures or an analysis-of-covariance model. The mixed-effects model used the log-transformed postbaseline-to-baseline ratio as the dependent variable; treatment, visit, and their interaction (treatment × visit) as fixed effects; and the log-transformed baseline value of the corresponding endpoint as a covariate. An unstructured covariance model was used for within-patient errors across treatments, and SEs and tests were adjusted for small samples using the Kenward-Roger approach. The analysis-of-covariance model also used the log-transformed postbaseline-to-baseline ratio as the dependent variable, with treatment as a fixed effect and the log-transformed baseline value as a covariate. Results are presented as the percentage change with 95% CI, estimated using the exponentiated least squares mean estimate: percentage change = (exp[β] − 1) × 100. The normality assumption was assessed using the Shapiro-Wilk test of the studentized residuals from the fitted model.

The correlations of Lp(a)-C, Lp(a)-TG, and Lp(a) with other lipid analytes at baseline were assessed using Spearman’s correlation coefficient (*r*). The correlations of these variables with other lipid analytes at the primary analysis time point were assessed using partial Spearman’s correlation coefficient (*r*), controlling for treatment and log-transformed baseline values. Given the exploratory nature of the analyses conducted in support of this publication, no multiple-comparison procedure or multiplicity adjustment was applied to control the overall type I error rate.

Statistical analyses were performed using SAS version 9.4 (SAS Institute), and a *P* value <0.05 was considered to indicate nominal significance.

## Results

### Baseline laboratory variables in the phase 2 clinical trial

Baseline lipid and lipoprotein characteristics were comparable across placebo and olezarsen treatment groups (Table 1). Participants exhibited moderate hypertriglyceridemia with similar apoC-III concentrations; overlapping distributions of total cholesterol, LDL cholesterol, non–high-density lipoprotein (HDL) cholesterol, and HDL cholesterol; and comparable apoB levels (∼77-89 mg/dL [1,504-1,725 nmol/L]). Median Lp(a) concentrations showed wide interindividual variability but did not differ systematically across groups.

**Table 1:** Baseline Laboratory Variables

	Placebo (n = 24)	OLE 10 mg Q4W (n = 22)	OLE 15 mg Q2W (n = 23)	OLE 10 mg QW (n = 23)	OLE 50 mg Q4W (n = 22)
Cumulative monthly dose, mg		10	30	40	50
Triglycerides, mg/dL	293.8 ± 86.7	281.6 ± 73.4	284.8 ± 94.6	292.3 ± 89.3	268.4 ± 85.1
ApoC-III, mg/dL	16.6 ± 4.5	16.0 ± 4.2	15.9 ± 4.3	16.8 ± 4.2	15.7 ± 3.3
TC, mg/dL	144.8 ± 26.0	149.2 ± 28.8	163.1 ± 35.1	160.0 ± 34.6	166.8 ± 35.3
Derived VLDL-C, mg/dL	53.6 ± 11.7	53.9 ± 12.7	57.1 ± 24.7	53.5 ± 12.1	56.6 ± 26.7
LDL-C by ultracentrifugation, mg/dL	60.2 ± 27.2	61.6 ± 18.9	70.5 ± 23.2	76.2 ± 30.5	76.8 ± 20.8
LDL-C by ultracentrifugation, corrected for Lp(a)-C, mg/dL	53.0 ± 25.0	51.3 ± 20.9	63.0 ± 25.5	67.4 ± 25.9	70.5 ± 20.8
HDL-C, mg/dL	34.6 ± 8.6	34.1 ± 9.1	33.9 ± 9.5	34.8 ± 8.6	36.8 ± 10.5
Non-HDL-C, mg/dL	110.3 ± 24.0	115.1 ± 28.9	129.2 ± 33.3	125.3 ± 32.1	130.1 ± 31.0
ApoAI, mg/dL	131.4 ± 19.3	130.2 ± 19.2	129.0 ± 16.7	132.5 ± 23.0	136.3 ± 23.1
ApoB, mg/dL	77.1 ± 19.7	79.6 ± 16.6	87.9 ± 20.6	86.8 ± 19.6	88.5 ± 14.3
ApoB, nmol/L	1,504 ± 384	1,552 ± 323	1,713 ± 401	1,691 ± 382	1,725 ± 279
ApoB, mg/dL	80.8 ± 31.0	80.3 ± 15.5	85.5 ± 29.5	84.0 ± 26.0	91.8 ± 9.5
Lp(a), nM	17.0 ± 33.3	37.3 ± 56.5	25.5 ± 65.0	33.0 ± 129.5	20.0 ± 57.0
Non-Lp(a)-apoB^a^, nM	1,558 ± 638	1,528 ± 459	1,641 ± 640	1,604 ± 636	1,769 ± 242
OxPL-apoB, nM	1.53 (1.00-1.80)	1.43 (0.67-7.25)	1.70 (1.03-4.47)	1.86 (1.04-2.81)	1.59 (1.16-2.82)
OxPL-apo(a), nM	1.21 (0.29-2.38)	3.72 (0.66-39.68)	1.64 (0.46-8.50)	3.34 (0.69-5.71)	1.18 (0.63-12.35)
OxPL-apoC-III, nM	1.21 (0.60-2.06)	1.40 (0.88-2.25)	1.49 (1.34-1.78)	1.35 (0.99-1.93)	1.85 (1.30-2.42)
OxPL-apoAI, nM	0.39 (0.14-0.68)	0.28 (0.16-0.59)	0.35 (0.12-0.63)	0.59 (0.09-0.71)	0.37 (0.17-0.72)

Values are mean ± SD or median (Q1-Q3). Derived VLDL-C is calculated as VLDL-C = TC − LDL-C by ultracentrifugation − HDL-C.

apoAI = apolipoprotein AI; apo(a) = apolipoprotein(a); apoB = apolipoprotein B; apoC-III = apolipoprotein C-III; HDL-C = high-density lipoprotein cholesterol; LDL-C = low-density lipoprotein cholesterol; Lp(a) = lipoprotein(a); Lp(a)-C = lipoprotein(a)-associated cholesterol; OLE = olezarsen; OxPL = oxidized phospholipids; Q2W = every 2 weeks; Q4W = every 4 weeks; QW = every week; TC = total cholesterol; VLDL-C = very low density lipoprotein cholesterol.

a Non-Lp(a)-apoB values were derived by converting apoB to molar concentration by multiplying apoB mass in milligrams per deciliter by 19.49 using a nonglycosylated molecular weight of 513,000 and then subtracting Lp(a)-apoB.^26^

### Correlation analyses

Lp(a)-TG showed nonsignificant correlations with apoB (*r* = 0.16; *P* = 0.17). In contrast, Lp(a)-C exhibited positive correlations with apoB (*r* = 0.31; *P* = 0.006). Spearman correlations of OxPL parameters are shown in Table 2. OxPL-apoB showed a weak association with Lp(a)-C but not with Lp(a)-TG. OxPL-apo(a) showed a strong positive correlation with Lp(a) (*r* = 0.70; *P* < 0.001), paralleled by OxPL-apoB (*r* = 0.59; *P* < 0.001), confirming Lp(a) as the dominant carrier of circulating OxPL. OxPL-apoAI also demonstrated a significant but more modest association with Lp(a) (*r* = 0.36; *P* < 0.001). There was no correlation of apoC-III or triglycerides with any of the OxPL measures.

**Table 2:** Spearman Correlations Between OxPL on Apolipoprotein Carriers and Lipid Parameters at Baseline

	OxPL-ApoB	OxPL-Apo(a)	OxPL-ApoC-III	OxPL-ApoAI
Lp(a)-C, mg/dL	0.23^a^	0.18	−0.04	0.18
Lp(a)-TG, mg/dL	−0.07	0.06	0.02	0.17
ApoC-III, mg/dL	−0.02	−0.06	0.09	0.05
Lp(a), nmol/L	0.59^b^	0.70^b^	0.18	0.36^b^
Total cholesterol, mg/dL	−0.02	−0.13	0.16	−0.02
Triglycerides, mg/dL	0.03	−0.08	0.10	0.05
ApoB, mg/dL	0.01	−0.04	0.20	−0.05
LDL-C by ultracentrifugation, mg/dL	−0.02	−0.06	0.13	−0.02
Corrected LDL-C by ultracentrifugation, mg/dL	−0.25^a^	−0.28^c^	0.09	−0.11
HDL-C, mg/dL	−0.01	0.05	0.08	−0.01
Derived VLDL-C, mg/dL	0.01	−0.11	0.06	−0.00
Non-HDL-C, mg/dL	−0.02	−0.15	0.13	−0.02
ApoAI, mg/dL	0.02	0.02	0.03	0.01
Total apoB, nmol/L	0.01	−0.04	0.20	−0.05
Non-Lp(a)-apoB, nmol/L	−0.14	−0.18	0.21^a^	−0.10

Lp(a)-C = lipoprotein(a)-associated triglycerides; other abbreviations as in Table 1.

a *P* < 0.05.

b *P* < 0.001.

c *P* < 0.01.

Both OxPL-apo(a) and OxPL-apoB were inversely associated with corrected LDL cholesterol, including ultracentrifugation-derived measures (*r* = −0.25 to −0.28; *P* < 0.05 for all), indicating that OxPL burden is not directly proportional to LDL cholesterol content. OxPL-apoC-III demonstrated a modest positive association with non-Lp(a)-apoB (*r* = 0.21; *P* = 0.040).

No significant correlations were observed between OxPL measures and total apoB, VLDL, or non-HDL cholesterol concentrations.

### Effects of olezarsen on Lp(a)-associated lipids and ratios

**Figure 2:**
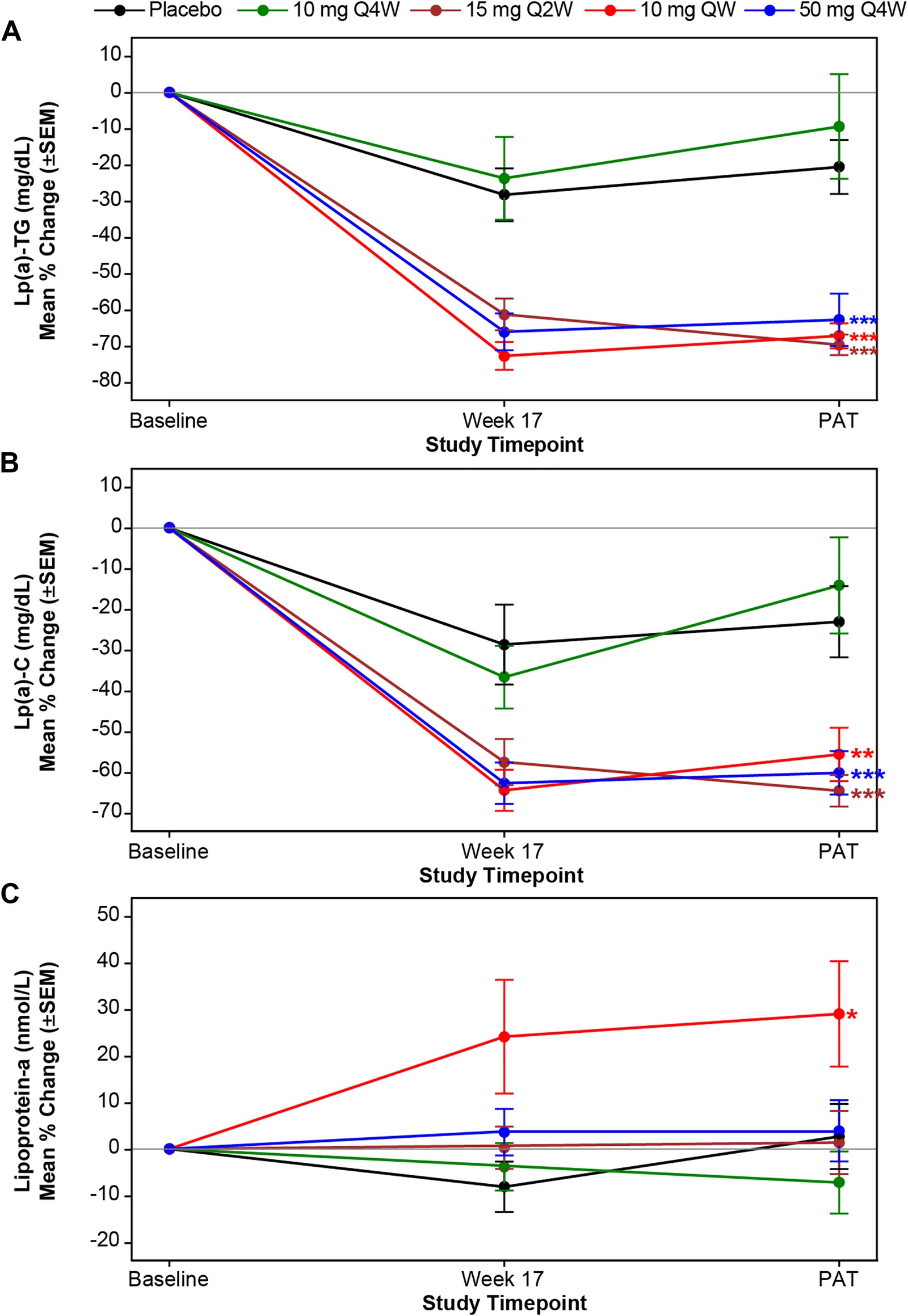
Temporal Changes in Lp(a)-TG, Lp(a)-C, and Lp(a) in Response to Olezarsen Plasma (A) Lp(a)-associated triglycerides (Lp[a]-TG), (B) Lp(a)-associated cholesterol (Lp[a]-C), and (C) Lp(a) molar concentrations were measured at baseline and at the primary analysis time point (PAT) in placebo- and olezarsen-treated participants. Data are shown for the different olezarsen dosing regimens. Values represent mean percentage change from baseline ± SEM. ∗*P* = 0.05, ∗∗*P* = 0.01, and ∗∗∗*P* < 0.001 at the primary analysis time point compared with placebo. Q2W = every 2 weeks; Q4W = every 4 weeks; QW = every week.

At baseline, Lp(a)-TG and Lp(a)-C were readily detectable across all groups, with Lp(a)-TG/Lp(a)-C ratios ranging from 3.8 to 4.1 (Table 3). Olezarsen treatment produced marked, dose-dependent reductions in Lp(a)-TG, with decreases of 62.9% to 69.8% at the higher cumulative monthly doses (30-50 mg; *P* < 0.001 vs placebo), compared with minimal change in the placebo and lowest dose group (Figure 2A). Lp(a)-C was similarly reduced by 55.7% to 64.6% at higher doses (*P* = 0.005 vs placebo) (Figure 2B). This did not result in consistent reductions in total cholesterol or corrected LDL-C measured by ultracentrifugation across dose regimens (Table 3).

**Table 3:** Baseline and Changes at the Primary Analysis Time Point of Lp(a)-TG, Lp(a)-C, Lp(a), and Lp(a)-TG/Lp(a)-C Ratio

	Placebo	OLE 10 mg Q4W	OLE 15 mg Q2W	OLE 10 mg QW	OLE 50 mg Q4W
Cumulative monthly dose, mg		10	30	40	50
Lp(a)-TG, mg/dL					
Baseline	59.1 ± 31.0	46.2 ± 20.2	59.9 ± 25.9	54.2 ± 22.4	48.7 ± 24.9
% of plasma triglyceride	20.4 ± 7.3	16.0 ± 5.0	21.4 ± 7.5	18.3 ± 6.4	18.8 ± 8.6
Primary endpoint	46.1 ± 25.7	31.9 ± 17.2	17.2 ± 7.2	15.5 ± 6.6	14.5 ± 8.2
% change	−20.6 ± 31.7	−9.4 ± 56.1	−69.8 ± 11.3	−67.3 ± 13.4	−62.9 ± 31.6
*P* value vs placebo		0.86	<0.001	<0.001	<0.001
Lp(a)-C, mg/dL					
Baseline	15.2 ± 8.1	12.8 ± 5.6	15.5 ± 6.8	14.8 ± 7.3	13.7 ± 6.7
% of plasma total cholesterol	10.7 ± 5.6	8.6 ± 3.4	9.8 ± 4.5	9.7 ± 4.1	8.6 ± 5.0
Primary endpoint	11.9 ± 7.0	8.8 ± 5.1	5.2 ± 2.7	6.1 ± 4.1	5.2 ± 3.8
% change	−23.1 ± 37.2	−14.1 ± 45.7	−64.6 ± 15.6	−55.7 ± 25.4	−60.2 ± 23.2
*P* value vs placebo		0.54	<0.001	0.005	<0.001
Lp(a), nmol/L					
Baseline	58.9 ± 90.8	86.0 ± 81.2	62.2 ± 75.1	72.8 ± 101.7	51.4 ± 71.0
Primary endpoint	62.0 ± 97.6	92.4 ± 92.0	57.6 ± 68.7	67.1 ± 80.3	49.5 ± 61.2
% change	+2.7 ± 31.3	−7.2 ± 28.4	+1.4 ± 29.6	+29.1 ± 48.1	+3.9 ± 30.2
*P* value vs placebo		0.30	0.92	0.040	0.91
Lp(a), nmol/L					
Baseline	17.0 (9.0-42.3)	37.3 (14.0-170.5)	25.5 (13.0-78.0)	33.0 (9.0-138.5)	20.0 (13.0-70.0)
Primary endpoint	18.0 (8.5-44.5)	56.5 (14.0-179.0)	25.0 (18.0-65.0)	32.5 (18.0-80.0)	20.0 (13.0-75.0)
% change	0.0 (−18.2 to 18.5)	−6.5 (−24.5 to 7.1)	−1.0 (−16.7 to 13.8)	9.1 (−7.7 to 52.9)	0.00 (−15.0 to 23.8)
*P* value vs placebo		0.30	0.92	0.040	0.91
Lp(a)-TG/Lp(a)-C ratio					
Baseline	4.0 ± 0.9	3.9 ± 1.3	4.1 ± 1.2	4.1 ± 1.6	3.8 ± 1.1
Primary endpoint	4.3 ± 1.6	3.8 ± 1.6	3.7 ± 1.4	3.3 ± 1.6	3.4 ± 1.9
% change	+10.3 ± 35.6	+5.6 ± 49.3	+7.6 ± 33.0	−17.7 ± 30.2	−9.7 ± 41.9
*P* value vs placebo		0.41	0.18	0.029	0.030
Lp(a)-TG/Lp(a) ratio					
Baseline	46.5 ± 41.5	22.0 ± 23.7	35.3 ± 32.6	31.3 ± 27.1	25.6 ± 20.2
Primary endpoint	33.3 ± 39.0	20.2 ± 26.8	9.4 ± 8.7	6.2 ± 4.0	10.6 ± 13.5
% change	−13.3 ± 41.5	+24.7 ± 128.0	−65.1 ± 16.3	−71.1 ± 15.4	−58.7 ± 43.7
*P* value vs placebo		0.94	0.002	<0.001	<0.001
Lp(a)-C/Lp(a) ratio					
Baseline	26.0 ± 23.8	11.6 ± 12.5	19.9 ± 18.5	17.5 ± 15.7	16.6 ± 14.5
Primary endpoint	17.7 ± 24.6	9.8 ± 12.3	5.7 ± 4.3	4.2 ± 2.7	7.8 ± 15.1
% change	−19.6 ± 37.1	+5.7 ± 64.7	−62.5 ± 18.3	−59.5 ± 29.6	−57.9 ± 27.9
*P* value vs placebo		0.89	<0.001	<0.001	<0.001
Total cholesterol, mg/dL					
Baseline	144.8 ± 26.0	149.2 ± 28.8	163.1 ± 35.1	160.0 ± 34.6	166.8 ± 35.3
Primary endpoint	148.6 ± 29.1	148.7 ± 30.3	142.7 ± 27.4	150.9 ± 30.5	153.3 ± 42.2
% change	3.0 ± 14.5	0.1 ± 18.0	−10.9 ± 12.2	−1.8 ± 14.4	−8.0 ± 12.9
*P* value vs placebo		0.57	0.008	0.45	0.061
Corrected LDL-C by ultracentrifugation, mg/dL					
Baseline	53.0 ± 25.0	51.3 ± 20.9	63.0 ± 25.5	67.4 ± 25.9	70.5 ± 20.8
Primary endpoint	51.4 ± 25.6	56.8 ± 29.6	63.7 ± 27.9	74.0 ± 25.1	71.4 ± 22.5
% change	−1.9 ± 25.7	−0.1 ± 38.4	1.7 ± 19.1	30.8 ± 62.5	13.3 ± 34.0
*P* value vs placebo		0.83	0.67	0.003	0.20
ApoB, mg/dL					
Baseline	77.1 ± 19.7	79.6 ± 16.6	87.9 ± 20.6	86.8 ± 19.6	88.5 ± 14.3
Primary endpoint	75.9 ± 17.7	83.2 ± 18.4	71.3 ± 17.9	79.1 ± 15.7	76.8 ± 16.4
% change	1.3 ± 13.5	1.4 ± 15.9	−17.0 ± 12.6	−5.2 ± 19.5	−12.2 ± 13.5
*P* value vs placebo		0.68	<0.001	0.32	0.024
HDL-C, mg/dL					
Baseline	34.6 ± 8.6	34.1 ± 9.1	33.9 ± 9.5	34.8 ± 8.6	36.8 ± 10.5
Primary endpoint	35.8 ± 9.7	38.4 ± 13.3	46.8 ± 16.0	46.6 ± 12.3	47.5 ± 12.9
% change	−0.3 ± 13.1	12.6 ± 21.1	34.8 ± 21.9	42.3 ± 21.1	30.7 ± 22.2
*P* value vs placebo		0.037	<0.001	<0.001	<0.001

Values are mean ± SD or median (Q1-Q3). *P* values were obtained using an MMRM or an ANCOVA model. The MMRM included the log-transformed postbaseline-to-baseline ratio as the dependent variable; treatment, visit, and the treatment-by-visit interaction as fixed effects; and the log-transformed baseline value of the corresponding endpoint as a covariate. The ANCOVA model included the log-transformed postbaseline-to-baseline ratio as the dependent variable, treatment as a fixed effect, and the log-transformed baseline value of the corresponding endpoint as a covariate.

ANCOVA = analysis of covariance; MMRM = mixed-effects model for repeated measures; other abbreviations as in Tables 1 and 2.

**Figure 3:**
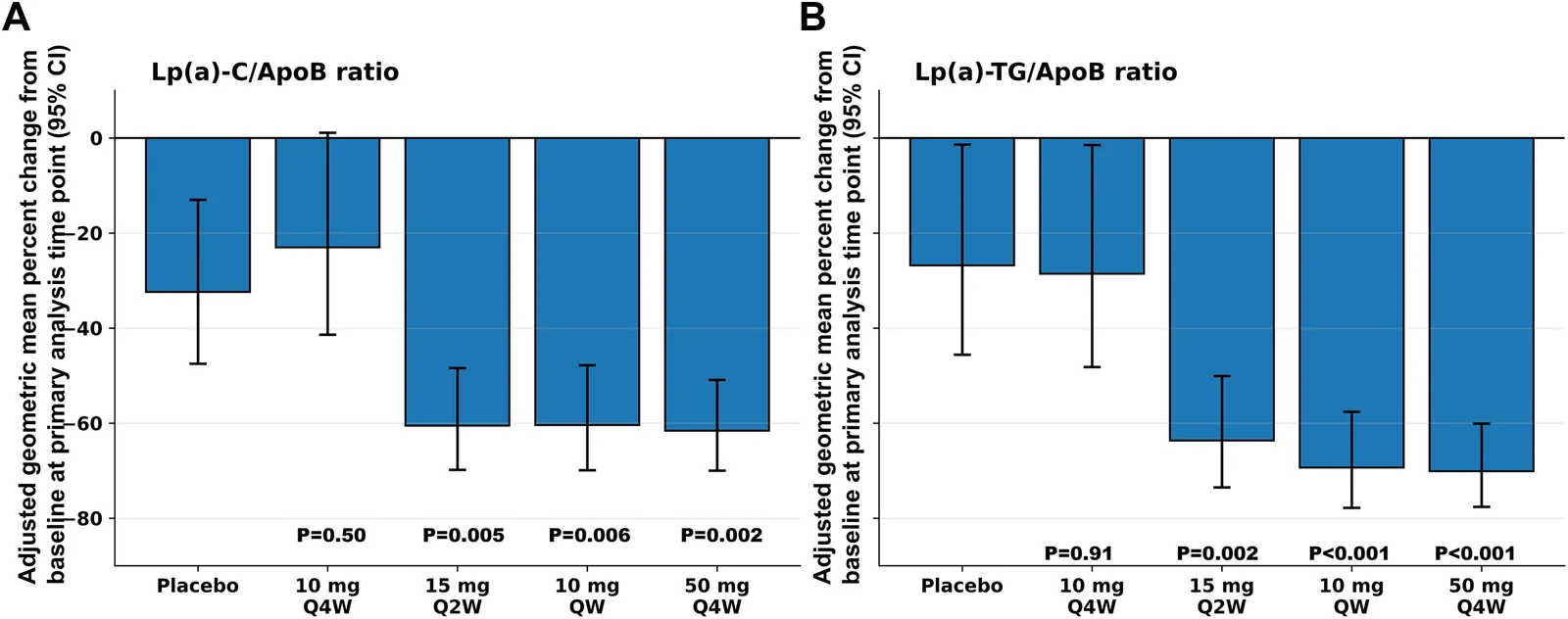
Effects of Olezarsen on ApoB-Normalized Lp(a) Lipid Ratios at the Primary Analysis Time Point (A) Adjusted geometric mean percentage change from baseline in the Lp(a)-C/apoB ratio and (B) adjusted geometric mean percentage change from baseline in the Lp(a)-TG/apoB ratio in placebo- and olezarsen-treated participants with moderate hypertriglyceridemia. Data are shown for the primary analysis time point (week 25 for Q4W dosing cohorts and week 27 for Q2W and QW dosing cohorts). Error bars represent asymmetrical 95% CIs. *P* values are vs pooled placebo. Abbreviations as in Figures 1 and 2.

To determine whether reductions in Lp(a)-associated lipids reflected generalized lowering of apoB-containing lipoproteins or selective remodeling of Lp(a), apoB-normalized compositional ratios were evaluated. For the Lp(a)-C/apoB ratio, placebo-adjusted reductions reached 60.5% to 61.6% at the higher clinically effective doses (15 mg every 2 weeks, 10 mg every week, and 50 mg every 4 weeks; *P* = 0.005-0.002) (Figure 3A). Similarly, the Lp(a)-TG/apoB ratio decreased by 63.7% to 70.1% at these same doses (*P* = 0.002 to <0.001) (Figure 3B). In complementary mixed-effect models, the ratio of Lp(a)-TG to corrected LDL cholesterol demonstrated percentage reductions in adjusted geometric means of 67.8% to 77.4% (*P* < 0.001), while the ratio of Lp(a)-C to corrected LDL cholesterol decreased by 57.2% to 68.9% (*P* = 0.002 to <0.001) at the 3 highest olezarsen doses at the primary analysis time point.

### Lipoprotein size fractionation results in placebo vs olezarsen

**Figure 4:**
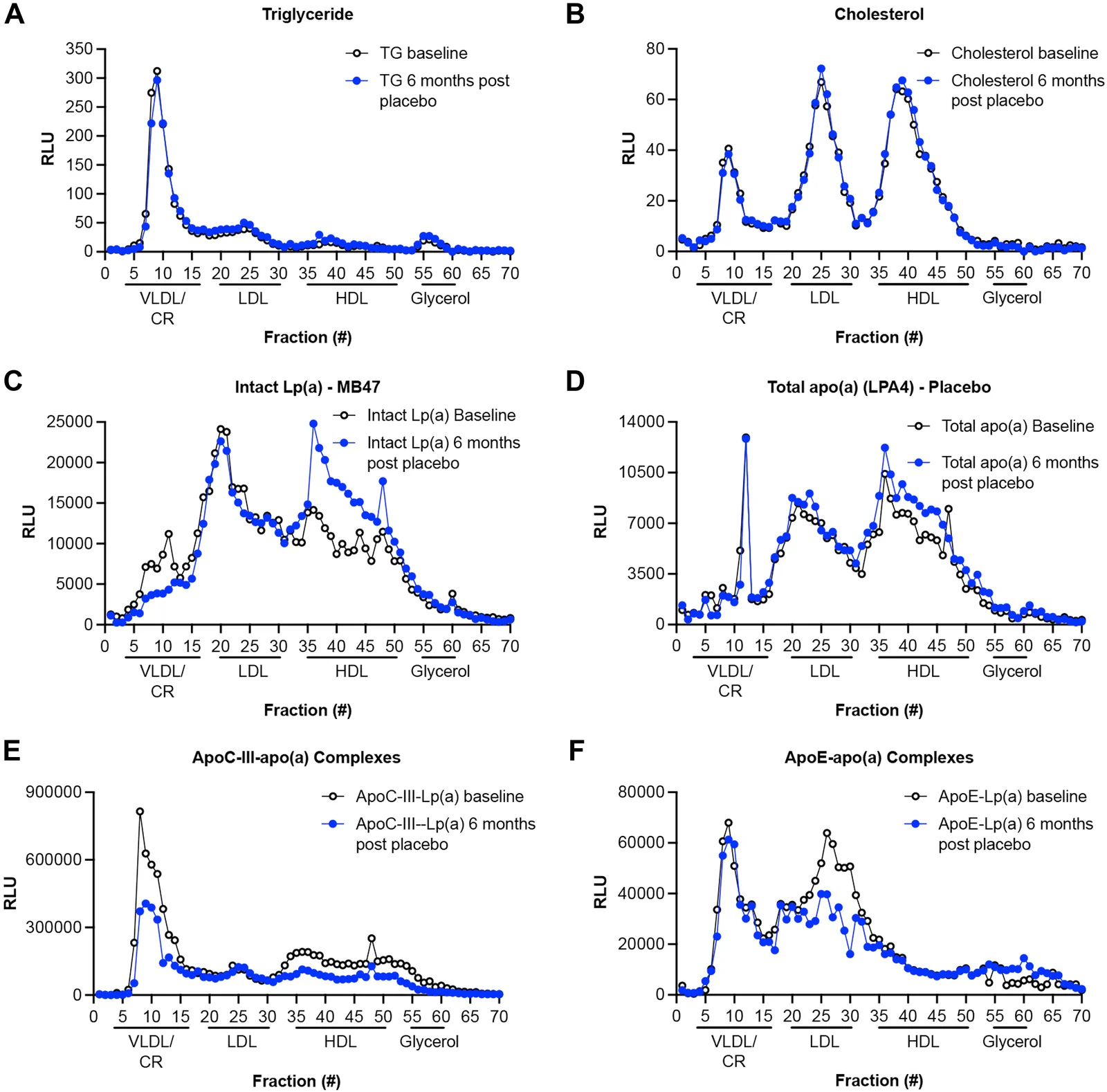
FPLC Distribution of Triglycerides, Cholesterol, Intact Lp(a), Total Apo(a), and Apo(a)-Containing Particles in Placebo-Treated Participants Size distribution of lipids and apo(a)-containing particles in placebo-treated participants. Fast protein liquid chromatography (FPLC) mean profiles from 5 participants showing plasma (A) triglycerides, (B) cholesterol, (C) intact Lp(a), (D) total apo(a), (E) apoC-III-Lp(a) complexes, and (F) apolipoprotein E (apoE)–Lp(a) complexes are shown at baseline and after 6 months of placebo treatment. (A) Triglycerides localized predominantly to VLDL fractions, whereas cholesterol was enriched in LDL- and HDL-sized fractions. Intact Lp(a) and total apo(a) were distributed primarily within LDL-sized fractions. ApoC-III-Lp(a) complexes were enriched in triglyceride-rich fractions, while apoE-Lp(a) complexes were detected mainly in VLDL and LDL fractions. These distributions were largely unchanged after 6 months of placebo treatment, indicating the stability of lipoprotein and apo(a)-containing particle profiles over time. Abbreviations as in Figures 1 and 2.

In placebo-treated participants, plasma triglycerides were predominantly distributed within VLDL fractions (fractions ∼5-15) (Figure 4A), whereas cholesterol was enriched in LDL-sized fractions (fractions ∼20-30) with a secondary peak in HDL fractions (fractions ∼35-50) (Figure 4B). Intact Lp(a), measured by apoB-100 capture, was localized primarily to larger sized LDL fractions, with a smaller signal extending into adjacent smaller HDL-sized ranges (Figure 4C). Total apo(a) showed a similar distribution, with a small peak signal in VLDL fractions (Figure 4D). ApoC-III-Lp(a) complexes were enriched in triglyceride-rich fractions and minimally present across LDL-sized fractions (Figure 4E), whereas apoE-Lp(a) complexes were detected primarily in VLDL and LDL fractions (Figure 4F). At weeks 25 to 27, these distributions were largely unchanged in the placebo group, indicating stability of lipoprotein and apo(a)-containing particle profiles over time. This glycerol fraction (fractions ∼52-60) was unaltered in the placebo control group.

**Figure 5:**
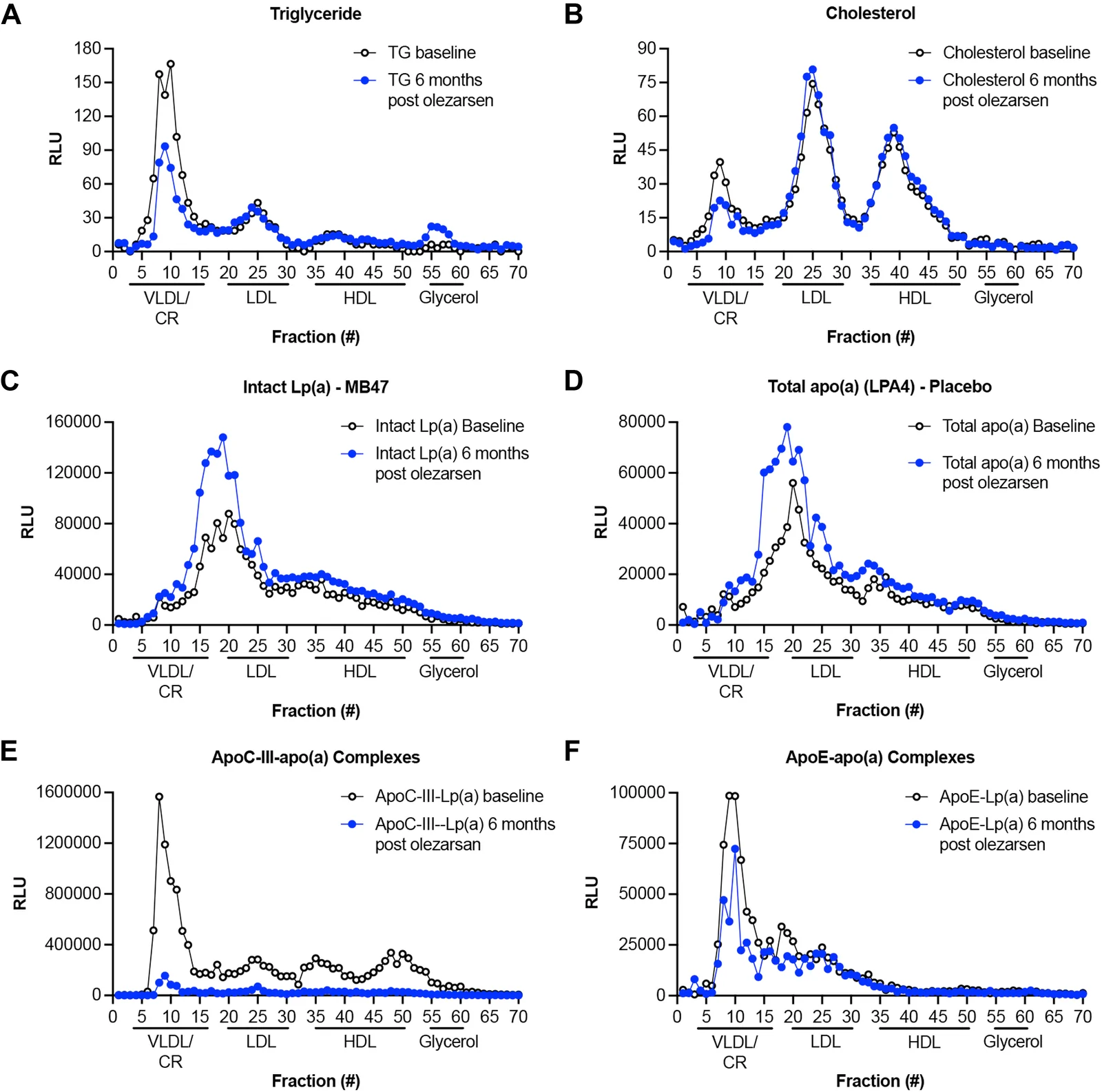
FPLC Distribution of Triglycerides, Cholesterol, Intact Lp(a), Total Apo(a) and Apo(a)-Containing Particles in Olezarsen-Treated (50 mg) Participants Size distribution of lipids and apo(a)-containing particles in olezarsen-treated (50 mg group) participants. FPLC mean profiles from 5 participants showing plasma (A) triglycerides, (B) cholesterol, (C) intact Lp(a), (D) total apo(a), (E) apoC-III-Lp(a) complexes, and (F) apoE-Lp(a) complexes are shown at baseline and after 6 months of olezarsen treatment. Olezarsen markedly reduced triglycerides and cholesterol in VLDL fractions, with preservation of LDL-sized intact Lp(a) and total apo(a) distributions. ApoC-III-Lp(a) complexes were substantially reduced across the lipoprotein distribution, whereas apoE-Lp(a) complexes showed reduction within triglyceride-rich fractions. Abbreviations as in Figures 1, 2, and 4.

In participants treated with olezarsen 50 mg, baseline distributions of triglycerides, cholesterol, intact Lp(a), total apo(a), and apo(a)-associated apolipoproteins were similar to those observed in the placebo group. Following 6 months of olezarsen 50 mg treatment, triglycerides were markedly reduced in VLDL fractions, with minimal change in LDL- and HDL-sized fractions (Figure 5A). The glycerol fraction was elevated after olezarsen 50 mg treatment, which is indicative of increased lipoprotein lipase activity and particle remodeling. Cholesterol levels showed a reduced signal in VLDL fractions without changes in LDL- and HDL-sized fractions (Figure 5B). Intact Lp(a) and total apo(a) distributions were preserved or increased in LDL-sized fractions (Figures 5C and 5D). In contrast, apoC-III-Lp(a) complexes were substantially reduced across the lipoprotein distribution (Figure 5E), whereas apoE-Lp(a) complexes showed reduction primarily within triglyceride-rich fractions (Figure 5F).

Quantitative analysis of the individual FPLC profiles from 5 placebo-treated and 5 olezarsen 50 mg–treated participants demonstrated that apoC-III inhibition with olezarsen substantially reduced triglyceride signal across the FPLC profile. The greatest reduction was observed in the early, larger TRL fractions (fractions 1-12; −54.3% ± 35.1%), whereas a more modest reduction was observed in later LDL-sized fractions (fractions 15–30; −18.6% ± 18.8%), resulting in an overall reduction of −34.1% ± 30.9%. Cholesterol demonstrated a similar pattern, with marked reductions in early TRL fractions (−54.6% ± 28.0%) and little overall change in LDL-sized fractions (+8.9% ± 17.6%). Consistent with these findings, apoC-III-associated Lp(a) signal was reduced by approximately 70% to 75% across early, LDL-sized, and overall fractions, whereas intact Lp(a) and total apo(a) signals were generally preserved despite substantial interindividual variability (Supplemental Table 2).

Relative distribution analyses demonstrated that olezarsen reduced the proportion of triglycerides within TRL fractions from 60.7% to 38.6%, with reciprocal enrichment of non-TRL fractions from 39.3% to 61.4% (Supplemental Table 3). Cholesterol distribution similarly shifted away from TRL fractions (17.7%-11.2%) toward non-TRL fractions (82.3%-88.8%). In contrast, the proportional distribution of intact Lp(a) and total apo(a) between TRL and non-TRL fractions changed minimally following treatment.

### Effect of olezarsen on OxPL parameters

At the primary analysis time point, changes in OxPL measures were variable and not significantly different from placebo across treatment groups despite substantial reductions in Lp(a)-TG and Lp(a)-C (Supplemental Table 4). OxPL-apo(a) and OxPL-apoB demonstrated numerically increased adjusted geometric mean percent changes in several olezarsen dose groups, whereas OxPL-apoC-III and OxPL-apoAI showed smaller and less consistent changes.

### Effects of olezarsen on Lp(a)-associated lipid composition and OxPL measures

Among participants with elevated baseline Lp(a) (≥75 nmol/L), significant reductions in Lp(a)-C were observed with the 3 highest cumulative olezarsen doses, whereas significant reductions in Lp(a)-TG were observed with 15 mg every 2 weeks, 10 mg every week, and 50 mg every 4 weeks (Supplemental Figure 1).

Baseline OxPL and OxPL/Lp(a) ratio measures were further evaluated according to baseline tertiles of Lp(a)-C and Lp(a)-TG in pooled olezarsen-treated participants. Across increasing Lp(a)-C tertiles, OxPL-apo(a) and OxPL-apoB progressively increased, whereas OxPL-apoC-III remained relatively stable. OxPL-apo(a)/Lp(a) ratios showed a modest increase across Lp(a)-C tertiles, whereas OxPL-apoB/Lp(a) and OxPL-apoC-III/Lp(a) ratios remained stable or decreased slightly (Supplemental Figure 2).

In contrast, increasing baseline Lp(a)-TG tertiles were not associated with progressively greater OxPL burden. OxPL-apo(a), OxPL-apoB, and OxPL-apoC-III levels, as well as corresponding OxPL/Lp(a) ratios, remained relatively stable across Lp(a)-TG tertiles (Supplemental Figure 2).

## Discussion

This study provides new translational insights into the biology of Lp(a) in the setting of disturbed TRL metabolism. In individuals with moderate hypertriglyceridemia, Lp(a) particles were substantially enriched in both triglycerides and cholesterol, identifying a compositionally distinct Lp(a) phenotype that differs from the classically described LDL-like particle observed in normolipidemic states. Pharmacologic inhibition of apoC-III with olezarsen resulted in marked and dose-dependent depletion of both Lp(a)-TG and Lp(a)-C without reducing circulating Lp(a) particle concentration. These findings identify Lp(a) lipid composition as a therapeutically modifiable biochemical feature; whether such compositional remodeling has independent implications for cardiovascular risk remains to be determined.

The observed reduction in Lp(a)-TG is more consistent with preferential clearance of larger, triglyceride-rich, apoC-III-containing particles, with persistence or replacement by particles exhibiting a more typical LDL-sized profile rather than direct intraparticle remodeling. The differential behavior of OxPL-associated measures further suggests that triglyceride-rich and OxPL-rich Lp(a) phenotypes may represent partially distinct metabolic subspecies.

Lp(a) has been characterized primarily by particle concentration, with less emphasis on its lipid composition in different metabolic states. Prior studies using ultracentrifugation and qualitative immunodetection suggested that apo(a) can associate with TRLs in hypertriglyceridemic or postprandial conditions, but these approaches could not distinguish stable compositional features of Lp(a) from transient metabolic intermediates.^12^^,^^32^, ^33^, ^34^, ^35^, ^36^, ^37^, ^38^ By directly quantifying triglycerides and cholesterol associated with apo(a) covalently bound to apoB-100, the present study demonstrates that Lp(a) may be markedly lipid enriched in moderate hypertriglyceridemia.

The parallel reductions in Lp(a)-TG and Lp(a)-C observed with apoC-III inhibition provide insights into how Lp(a) composition is modified in hypertriglyceridemic states. If enhanced lipoprotein lipase–mediated hydrolysis were the sole mechanism,^39^ a preferential reduction in triglyceride content would be expected, with relative preservation of cholesterol. The concordant depletion of Lp(a)-TG and Lp(a)-C suggests that apoC-III inhibition may reduce the formation, persistence, or detectability of TG- and cholesterol-enriched apo(a)-containing particles, rather than reflecting triglyceride hydrolysis alone.

Lipoprotein fractionation demonstrated marked depletion of triglycerides, apoC-III, and apoE within TRL fractions following olezarsen treatment, with preservation of the LDL-sized distribution of intact Lp(a) and total apo(a), supporting the conclusion that apoC-III inhibition primarily alters the lipid environment of triglyceride-rich particles without redistributing intact Lp(a) across size fractions. These findings are consistent with preferential depletion of TG-rich, apoC-III-associated Lp(a)-related particles, with relative preservation of LDL-sized Lp(a) signal across fractions. An apparent increase in free and total apo(a) signal within LDL-sized fractions was observed in this subset; however, this likely reflects redistribution of apo(a)-associated signal from triglyceride-rich fractions rather than a true increase in circulating Lp(a), particularly given the lack of consistent changes in plasma Lp(a) concentration across treatment groups.

Of significant interest, it was noted in this study the Lp(a)-TG and Lp(a)-C values represented approximately 20% of total plasma triglycerides and 10% of total plasma cholesterol. These values are significantly higher than in subjects with normal triglycerides^13^ or Lp(a).^25^ Although Lp(a) particle size was not directly measured, the observed enrichment of triglycerides relative to cholesterol is consistent with the presence of compositionally distinct, triglyceride-enriched Lp(a) particles in hypertriglyceridemic states. This interpretation is further supported by the high Lp(a)-TG/Lp(a)-C ratio observed in the present study, which contrasts with the lower triglyceride/cholesterol ratios typically seen in LDL particles under metabolically normal conditions.^40^^,^^41^ The contribution of triglyceride- and cholesterol-enriched Lp(a) particles to cardiovascular risk in hypertriglyceridemia remains unknown and warrants investigation in future studies.

The effects of therapies targeting *APOC3* messenger RNA on lipoprotein particle size and have not been extensively characterized with respect to molecular composition of Lp(a) particles. In a prior analysis from this trial, olezarsen was shown to produce dose-dependent shifts in nuclear magnetic resonance–derived triglyceride-rich, LDL, and HDL particle profiles, including reductions in TRL particles and redistribution of LDL and HDL subspecies.^42^ Similar changes in nuclear magnetic resonance–derived lipoprotein particle profiles have also been reported with plozasiran, a small interfering RNA targeting *APOC3*, supporting a class effect of apoC-III-lowering therapies on TRL metabolism.^43^ However, nuclear magnetic resonance methods cannot distinguish the molecular composition of particles of similar size, and because LDL and Lp(a) particles overlap in size, these approaches cannot specifically resolve Lp(a)-containing particles.

An important finding of this study is that OxPL burden demonstrated differential relationships with Lp(a) composition and apoC-III inhibition. OxPL-apo(a) and OxPL-apoB strongly correlated with Lp(a) particle concentration, confirming Lp(a) as the dominant lipoprotein carrier of circulating OxPL, whereas absolute Lp(a)-TG and Lp(a)-C concentrations showed only weak associations with OxPL measures. Baseline tertile analyses identified distinct cholesterol-rich/OxPL-enriched and triglyceride-rich/remodeled Lp(a) phenotypes. In addition, despite marked reductions in Lp(a)-TG and Lp(a)-C with olezarsen, OxPL measures were differentially affected across Lp(a)-associated compartments, with relative preservation of apo(a)-associated OxPL. Collectively, these findings support a model in which Lp(a) particle concentration, lipid composition, and associated OxPL cargo represent different dimensions of Lp(a) composition.

### Study limitations

Limitations of this study include that it was conducted in individuals with moderate hypertriglyceridemia, and the findings may not generalize to normotriglyceridemic populations. Samples were obtained in the fasting state, precluding assessment of postprandial dynamics, which may further accentuate apo(a)-TRL interactions. Free and esterified cholesterol could not be measured separately in the present study. In addition, the relationship between Lp(a) composition and apo(a) isoforms remains to be determined.

## Conclusions

In patients with moderate hypertriglyceridemia, inhibition of apoC-III with olezarsen results in marked reductions in triglyceride and cholesterol content on apo(a)-containing particles without reducing Lp(a) particle concentration. These findings demonstrate that Lp(a) lipid composition is dynamically modifiable in response to targeted therapy and support the concept that Lp(a) particle concentration, lipid composition, and associated OxPL cargo represent distinct associations of Lp(a) composition.

Perspectives **COMPETENCY IN MEDICAL KNOWLEDGE:** In patients with hypertriglyceridemia, Lp(a) particles exhibit distinct triglyceride-rich and OxPL-enriched compositional phenotypes compared with normolipidemic states. Inhibition of apoC-III with olezarsen markedly reduces Lp(a)-TG and cholesterol content without major reductions in Lp(a) particle concentration, demonstrating that Lp(a) lipid composition is dynamically modifiable and partially independent of particle number. OxPL measures tracked more closely with Lp(a) particle concentration than with Lp(a)-associated lipid content, with differential remodeling of apoC-III-associated and apo(a)-associated OxPL compartments, suggesting that Lp(a) particle concentration, lipid composition, and associated OxPL cargo represent partially distinct but biologically overlapping dimensions of Lp(a) biology.

**TRANSLATIONAL OUTLOOK:** These findings support a model in which Lp(a) composition is defined by particle number, lipid composition, and associated bioactive cargo such as OxPL. ApoC-III inhibition selectively remodels triglyceride-rich and apoC-III-associated Lp(a) components without major reductions in Lp(a) particle concentration, suggesting the presence of distinct metabolic Lp(a) subspecies. Future mechanistic and cardiovascular outcomes studies are needed to determine whether modification of Lp(a) composition influences cardiovascular risk independently of Lp(a) particle concentration and to identify which compositional features of Lp(a) are most closely linked to clinical events.

### Data Availability

Data requests from qualified researchers will be considered once all 3 of the following criteria are met: 1) 12 months from marketing approval of the study drug in both the United States and European Union; 2) 18 months from the conclusion of the study; and 3) 6 months from the publication of this paper. For additional information, visit https://vivli.org/ourmember/ionis/.

## Funding Support and Author Disclosures

This study was funded by Ionis Pharmaceuticals and by National Heart, Lung, and Blood Institute grants R01 HL159156 and HL170224 to Dr Tsimikas. Drs Alexander, Xia, and Tsimikas are employees of Ionis Pharmaceuticals. Dr Tsimikas is a coinventor of and receives royalties from patents owned by the University of California, San Diego; is a cofounder of and has an equity interest in Oxitope, Kleanthi Diagnostics, and Megaron; and has a dual appointment at the University of California, San Diego, and Ionis Pharmaceuticals. All other authors have reported that they have no relationships relevant to the contents of this paper to disclose.
