# Supplementary material for: A Novel Triglyceride- and Cholesterol-Enriched Lipoprotein(a) Phenotype in Moderate Hypertriglyceridemia: Reversal by ApoC-III Inhibition
Source: JACC Basic Transl Sci. 2026 Sep 17;11(10):101699. doi: 10.1016/j.jacbts.2026.101699 (PMC13602249; doi:10.1016/j.jacbts.2026.101699)
Supplement: Supplementary file 1 [file mmc1.docx]

**SUPPLEMENT**

**A Novel Triglyceride- and Cholesterol-Enriched Lipoprotein(a) Phenotype in Moderate Hypertriglyceridemia: Reversal by**

**ApoC-III Inhibition**

Lizhu Lin, PhD, Maiah N. Brush, BS, C. Bradley Nelson, BS,

Veronica J. Alexander, PhD, Shuting Xia, MS,

Philip L. S. M. Gordts, PhD, Sotirios Tsimikas, MD

**Supplementary Table 1**. Baseline laboratory variable of the 5 placebo and 5 olezarsen treated patients (mean ± SD) at Baseline and 6 Months.

LDL-C was measured with ultracentrifugation.

| **Timepoint** | **TC**  **(mg/dL)** | **LDL C (mg/dL)** | **TG**  **(mg/dL)** | **Lp(a) (nmol/L)** | **HDL-C (mg/dL)** | **APOC3 (mg/dL)** |
| --- | --- | --- | --- | --- | --- | --- |
| Placebo |  |  |  |  |  |  |
| Baseline | 146.4 ± 27.6 | 54.0 ± 32.6 | 309.4 ± 53.0 | 20.4 ± 14.8 | 36.0 ± 12.2 | 17.2 ± 3.2 |
| 6 months | 139.0 ± 32.7 | 50.8 ± 27.5 | 331.4 ± 139.3 | 21.6 ± 17.5 | 36.8 ± 14.0 | 17.0 ± 5.6 |
|  |  |  |  |  |  |  |
| Olezarsen 50 mg |  |  |  |  |  |  |
| Baseline | 163.8 ± 32.3 | 71.8 ± 29.2 | 287.0 ± 68.7 | 83.6 ± 93.0 | 33.2 ± 15.3 | 15.4 ± 3.6 |
| 6 months | 141.0 ± 40.6 | 73.0 ± 28.9 | 96.6 ± 31.6 | 75.2 ± 59.7 | 45.8 ± 22.2 | 3.0 ± 1.3 |

**Supplementary Table 2.** Changes in FPLC triglyceride, cholesterol, and Lp(a)-related measures with apoC-III inhibition with the olezarsen 50 mg dose.

| **Analyte** | **Region (fractions)** | Mean (± SD) % **change vs baseline** |
| --- | --- | --- |
| Triglycerides | 1–12 (early, larger/TRL) | −54.3 ± 35.1 |
| Triglycerides | 15–30 (LDL-sized) | −18.6 ± 18.8 |
| Triglycerides | 1–71 (overall) | −34.1 ± 30.9 |
| Cholesterol | 1–12 (early, larger/TRL) | −54.6 ± 28.0 |
| Cholesterol | 15–30 (LDL-sized) | +8.9 ± 17.6 |
| ApoC-III–Lp(a) | 1–12 (early) | −68.0 ± 33.1 |
| ApoC-III–Lp(a) | 15–30 (LDL-sized) | −75.5 ± 17.0 |
| ApoC-III–Lp(a) | 1–71 (overall) | −74.6 ± 20.8 |
| Intact Lp(a) | 1–71 (overall) | +33.2 ± 52.5 |
| Intact Lp(a) | 15–30 (LDL-sized) | +37.3 ± 39.1 |
| Total apo(a) | 1–71 (overall) | +63.6 ± 35.4 |
| Total apo(a) | 15–30 (LDL-sized) | +59.4 ± 35.1 |
| TG / intact Lp(a) ratio | 1–71 (overall) | −52.4 ± 25.8% |
| TG / intact Lp(a) ratio | 1–12 (early) | −66.8 ± 22.1% |

**Supplemental Table 3.** Relative Change Distribution in TRL Versus Non-TRL Triglyceride, Cholesterol, and Lp(a)-Related Measures with ApoC-III Inhibition with Olezarsen 50 mg Dose.

| **Placebo** | | | | | | |
| --- | --- | --- | --- | --- | --- | --- |
| Variable | **Baseline** | | **6 months treatment** | | **Change (%)** | |
|  | **% TRL** | **%**  **non-TRL** | **% TRL** | **%**  **non-TRL** | **TRL** | **non-TRL** |
| Lp(a) | 12.5 | 87.5 | 6.3 | 93.7 | -50% | 7% |
| Total apo(a) | 13.6 | 86.4 | 10.3 | 89.7 | -24% | 4% |
| ApoC-III-apo(a) | 39.5 | 60.5 | 35.5 | 64.5 | -10% | 7% |
| ApoE-apo(a) | 25.3 | 74.7 | 27.4 | 72.6 | 8% | -3% |
| Cholesterol | 14.9 | 85.1 | 14.2 | 85.8 | -5% | 1% |
| Triglycerides | 61.3 | 38.7 | 55.1 | 44.9 | -10% | 16% |
|  | | | | | | |
| **Olezarsen** | | | | | | |
| Variable | **Baseline** | | **6 months treatment** | | **Change (%)** | |
|  | **% TRL** | **%**  **non-TRL** | **% TRL** | **%**  **non-TRL** | **TRL** | **non-TRL** |
| Lp(a) | 13.7 | 86.3 | 15.7 | 84.3 | 15% | -2% |
| Total apo(a) | 15.6 | 84.4 | 15.9 | 84.1 | 2% | 0% |
| ApoC-III-apo(a) | 42.1 | 57.9 | 34.0 | 66.0 | -19% | 14% |
| ApoE-apo(a) | 54.6 | 45.4 | 46.2 | 53.8 | -15% | 19% |
| Cholesterol | 17.7 | 82.3 | 11.2 | 88.8 | -37% | 8% |
| Triglycerides | 60.7 | 39.3 | 38.6 | 61.4 | -36% | 56% |

**Supplemental Table 4.** Percent Changes in OxPL Measures with ApoC-III Inhibition.

| **Parameter** | **Treatment Group** | **Baseline, median (Q1-Q3), nM** | **Percent change**  **(95% CI)** | **P value vs placebo** |
| --- | --- | --- | --- | --- |
| OxPL-apoB | Placebo | 1.53 (1.00–1.80) | 9.15 (-22.2 to 53.1) | - |
|  | 10 mg Q4W | 1.43 (0.67–7.25) | 52.5 (7.8 to 115.6) | 0.17 |
|  | 15 mg Q2W | 1.70 (1.03–4.47) | 2.46 (-26.9 to 43.6) | 0.79 |
|  | 10 mg QW | 1.86 (1.04–2.81) | 32.3 (-7.3 to 88.8) | 0.44 |
|  | 50 mg Q4W | 1.59 (1.16–2.82) | 42.2 (1.6 to 99.1) | 0.27 |
| OxPL-apo(a) | Placebo | 1.21 (0.29–2.38) | -7.40 (-55.3 to 91.9) | - |
|  | 10 mg Q4W | 3.72 (0.66–39.68) | 55.1 (-26.7 to 228.1) | 0.33 |
|  | 15 mg Q2W | 1.64 (0.46–8.50) | 31.0 (-36.5 to 170.5) | 0.50 |
|  | 10 mg QW | 3.34 (0.69–5.71) | 107.2 (-3.5 to 344.7) | 0.13 |
|  | 50 mg Q4W | 1.18 (0.63–12.35) | 88.0 (-8.3 to 285.4) | 0.17 |
| OxPL-apoC-III | Placebo | 1.21 (0.60–2.06) | -15.9 (-43.0 to 24.2) | - |
|  | 10 mg Q4W | 1.40 (0.88–2.25) | -2.43 (-33.7 to 43.5) | 0.59 |
|  | 15 mg Q2W | 1.49 (1.34–1.78) | 2.75 (-29.5 to 49.6) | 0.47 |
|  | 10 mg QW | 1.35 (0.99–1.93) | -5.72 (-36.7 to 40.4) | 0.68 |
|  | 50 mg Q4W | 1.85 (1.30–2.42) | 14.1 (-22.7 to 68.4) | 0.28 |
| OxPL-apoAI | Placebo | 0.39 (0.14–0.68) | 60.9 (-3.1 to 167.3) | - |
|  | 10 mg Q4W | 0.28 (0.16–0.59) | 13.8 (-31.4 to 88.8) | 0.35 |
|  | 15 mg Q2W | 0.35 (0.12–0.63) | -22.0 (-53.7 to 31.5) | 0.051 |
|  | 10 mg QW | 0.59 (0.09–0.71) | 26.9 (-30.6 to 132.0) | 0.55 |
|  | 50 mg Q4W | 0.37 (0.17–0.72) | -2.76 (-39.0 to 55.1) | 0.15 |

Baseline values are presented as median (Q1-Q3). Percent changes and 95% CIs were estimated using MMRM at the primary analysis time point.

**Supplemental Figure 1. Percent change from baseline in Lp(a)-C and Lp(a)-TG at the primary analysis timepoint by baseline Lp(a) strata.** Mean percent changes from baseline in (A, C) Lp(a)-C and (B, D) Lp(a)-TG are shown for participants with baseline Lp(a) <75 nmol/L (top panels) and ≥75 nmol/L (bottom panels). Asterisks shown below each active-treatment bar denote the significance of the comparison versus pooled placebo using the pre-specified mixed model for repeated measures (MMRM) analysis: * P<0.05; **P<0.01; *** P<0.001*.* Values above or below each bar indicate the observed mean percent change. FAS = full analysis set (N=114).

**Supplemental Figure 2.** Baseline OxPL burden according to baseline Lp(a)-C and Lp(a)-TG tertiles. Baseline median (Q1–Q3) concentrations of OxPL-apo(a), OxPL-apoB, and OxPL-apoC-III, and the corresponding OxPL/Lp(a) ratios, in pooled olezarsen-treated participants (N=71) stratified by baseline Lp(a)-cholesterol [Lp(a)-C] tertiles (left) and baseline Lp(a)-triglyceride [Lp(a)-TG] tertiles (right). Higher Lp(a)-C tertiles were associated with progressively greater OxPL-apo(a) and OxPL-apoB levels, whereas higher Lp(a)-TG tertiles were not associated with progressive increases in OxPL burden. Values shown are medians (Q1–Q3) from pooled olezarsen groups. OxPL indicates oxidized phospholipid; apo(a), apolipoprotein(a); apoB, apolipoprotein B; apoC-III, apolipoprotein C-III; TG, triglycerides. Due to the exploratory nature of these analyses, formal statistical testing was not performed.
